# Emission of heavy metals from an urban catchment into receiving water and possibility of its limitation on the example of Lodz city

**DOI:** 10.1007/s10661-018-6648-9

**Published:** 2018-04-14

**Authors:** Grazyna Sakson, Agnieszka Brzezinska, Marek Zawilski

**Affiliations:** Institute of Environmental Engineering and Building Installations, Lodz University of Technology, Al. Politechniki 6, 90-924 Lodz, Poland

**Keywords:** Heavy metals, Stormwater management, Pollutant loads, Urban catchment

## Abstract

Heavy metals are among the priority pollutants which may have toxic effects on receiving water bodies. They are detected in most of samples of stormwater runoff, but the concentrations are very variable. This paper presents results of study on the amount of heavy metals discharged from urban catchment in Lodz (Poland) in 2011–2013. The research was carried out to identify the most important sources of their emission and to assess the threats to receiving water quality and opportunities of their limitation. The city is equipped with a combined sewerage in the center with 18 combined sewer overflows and with separate system in other parts. Stormwater and wastewater from both systems are discharged into 18 small urban rivers. There is a need of restoration of water bodies in the city. Research results indicate that the main issue is high emission of heavy metals, especially zinc and copper, contained in stormwater. Annual mass loads (g/ha/year) from separate system were 1629 for Zn and 305 for Cu. It was estimated that about 48% of the annual load of Zn, 38% of Cu, 61% of Pb, and 40% of Cd discharged into receiving water came from separate system, respectively 4% of Zn and Cu, 10% of Pb and 11% of Cd from CSOs, and the remaining part from wastewater treatment plant. Effective reduction of heavy metals loads discharged into receiving water requires knowledge of sources and emissions for each catchment. Obtained data may indicate the need to apply centralized solution or decentralized by source control.

## Introduction

The development of urbanized areas is associated with the emission of increased amounts of pollutants into the environment from built-up regions. This scenario also applies to pollutants discharged into surface waters from wastewater treatment plants, outfalls from drainage systems, combined storm overflows and surface runoff from the impervious area, and is particularly severe during wet weather. Requirements for discharges from wastewater treatment plants are generally precisely defined and strictly controlled. It is different in the case of outflows from separate systems. Requirements for the quality of stormwater runoff are differentiated in various countries, so the technical solutions adopted to meet them are also varied. In addition, monitoring of these outflows is difficult because of their usually high number on the urbanized area, as well as irregularity and unpredictability of the runoff and the multiplicity of pollutants. Stormwater is recognized as an important source of contaminants that may impact on receiving waters (Eriksson et al. 2007; Zghieb et al. 2012). They can get into rainwater during the formation of precipitation in the atmosphere, can be washed-off the catchment, and finally can originate from sewer deposit during conveyance by sewerage. More than 650 organic substances, 30 metals, and trace inorganic compounds may be contained in stormwater (Eriksson et al. 2007). Heavy metals are among components, which are the most commonly studied, especially zinc, copper, lead, and cadmium. They are detected in most of the samples of stormwater runoff, regardless of their place of collecting (Göbel et al. 2007; Gasperi et al. 2008; Zghieb et al. 2012). Sometimes they were detected in 100% of the stormwater runoff samples (Sabin et al. 2005). This is despite the restrictions introduced and action taken to reduce their use and emissions into the environment. The main sources of heavy metals in stormwater runoff are industries, buildings, particularly roofs with metal elements, vehicles’ parts and components, fuel and oils, and roads metallic structures (Gromaire et al. 2001; Brown and Peake 2006; Barbosa et al. 2012). The concentration of heavy metals vary considerably and mainly depend on the type of surface on which the rainfall occurs, the degree of contamination of the catchment area, and the characteristics of precipitation. Usually, the first flush phenomena is observed during stormwater runoff and CSO events (Tiefenthaler et al. 2008; Joshi and Balasubramanian 2010; Irvine et al. 2005).

Heavy metals are among the priority pollutants, which may have toxic effects on receiving water bodies and human health (Munch Christensen et al. 2006; Karlavičienė et al. 2009). According to Ma et al. (2016), even though the influence of a single heavy metal does not exert significant risk to people, the presence of multiple heavy metals could be toxic to human health with the contribution to the risk in the order: Pb > Cd > Cu > Zn. Although lead and cadmium are usually present in stormwater in lower concentrations than zinc and cooper, they pose higher risk. Heavy metals concentrations often exceed levels that are toxic to aquatic organisms (Selbig et al. 2013).

Effective protection of water bodies and ensuring their good ecological status established by European Water Framework Directive (EC 2000; EC 2008) requires, i.e., implementations of monitoring programs to gain the data necessary to prepare river basin management plan. An assessment of amount of pollutant loads and their impact on the aquatic environment are associated with wide and long-term studies, due to the high variability of the factors affecting their level. Additionally, as Borris et al. (2016) noticed, stormwater pollution is likely to change because of climate change, urbanization development, and environmental control. Hence, management of stormwater pollutant loads has become an important environmental concern (Becouze-Lareure et al. 2016) and predicting pollutants concentrations and loads has been one of the greatest challenges in urban hydrology over the last 20 years (Fletcher et al. 2013). Although new emerging contaminants in stormwater runoff are identified, heavy metals are still into focus (Gasperi et al. 2014; Becouze-Lareure et al. 2016; Kominková et al. 2016; Järveläinen et al. 2017), because despite many efforts their emission from urban catchment is still high. Blumensaat et al. (2012) who carried out water quality based assessment of urban drainage impacts in Europe, concluded that further fundamental research is required to better understand the relationships between urban wet weather discharges and stream ecosystem responses. Hence, there is a need to determine all sources of pollutants discharged from urban catchment into receiving water, including outflows from wastewater treatment plant, combined sewer overflows and stormwater runoff.

This paper presents results of research on the content of heavy metals in discharges from urban catchment, conducted in Lodz (Poland) in 2011–2013. The studies have been carried out in order to identify the most important sources of emissions of heavy metals and to assess the threats to receiving water quality and possibilities of their limitations. One of the purposes of this paper was to estimate the contributions of main sources of heavy metals, such as separated sewer system, combined sewer overflows, and wastewater treatment plant in total loads emitted from an urban catchment equipped with combined and separated sewerage.

## Materials and methods

### Study area

Lodz is one of the largest cities in Poland with 711.3 thousand inhabitants. It is located in the central part of the country on the border of two major rivers watersheds (Vistula and Oder). Elevation above sea level is between 163.6 and 284.1 m. Total area of the city is 293 km^2^ with structure of the land as follows: built-up and urbanized area—46.8%; agricultural land—41.7%; and forest land as well as woody and busty land—10.0%. Until the 1990s of the twentieth century in Lodz, there were many textile factories, so industrial effluents containing textile dyes were the major source of heavy metals. At present, there are only few textile factories in the city. Besides, there are factories of electro-mechanical, chemical, and leather-footwear industries, but because of development of in-house effluent treatment, industry is not recognized as a significant source of toxic metals. In Lodz, in areas outside the central districts, and especially in single-family home residential areas, roofs are usually covered with galvanized metal sheet or other metal elements. Previous research (Sakson 2017) indicated that runoff from roofs are often characterized by very high concentrations of zinc and copper due to the corrosion of metallic parts.

Lodz is in a temperate climate zone, with the average annual temperature of 8.0 °C, and monthly average from − 2.0 to 17.7 °C. The average annual precipitations (in the years 1904–2006) are from 364 to 808 mm with the annual average of 575 mm. On average, there are 167 days of wet weather per year (Podstawczynska 2010).

**Fig. 1:**
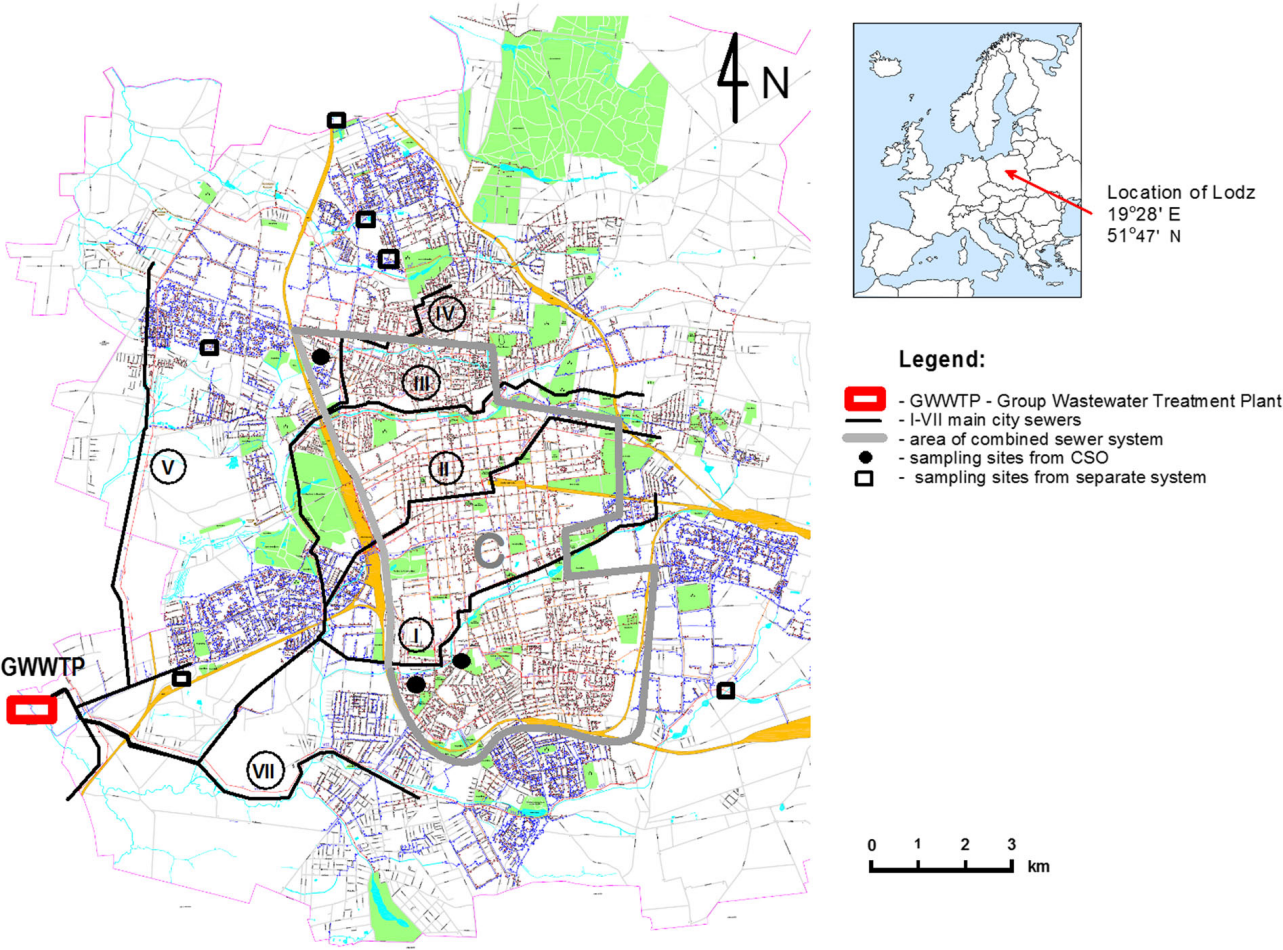
The scheme of Lodz city sewer system with sampling sites

Stormwater from the central parts of the city is directed to the combined sewer system and then to the municipal Group Wastewater Treatment Plant (WWTP) (Fig. 1). The biological stage of the WWTP works in the MUCT system. The average value of daily dry weather inflow amounts about 180,000 m^3^/day. However, the level of about 200,000 m^3^/day is exceeded during wet weather conditions, sometimes significantly. 99.9% of industrial and municipal wastewater in the city is treated. The catchment area of the combined sewerage is about 4240 ha. This system is equipped with 18 combined sewer overflows; on all, automatic flowmeters have been installed. Combined sewer overflows (CSOs) should function in Poland maximum ten times per year according to the Regulation of the Minister of Environment. However, they function much more frequently, causing a rapid discharge of untreated wastewater into surface waters (Table 1).

**Table 1:** Catchments of combined sewer system in the city center

River	Total catchment area (ha)	Number of CSOs	Number of overflows per year for single CSO	Total volume of overflows per year (thousand m^3^)
Jasień River	1910	7	4–48	528–932
Łódka River	920	8	7–51	127–270
Karolewka River	640	2	2–49	42–64
Bałutka River	550	1	6–27	75–260
Others	220			
Total	4240	18		

Other parts of the city are equipped with a separate sewer system. Its catchment area is about 6800 ha with the degree of imperviousness about 43%. Stormwater is discharged by numerous outlets to 18 small rivers; each of them has an average flow less than 1 m^3^/s (Table 2). Stormwater is discharged almost always without any pretreatment; settling tanks are located on just a few outlets. In outlying areas, which do not have any drainage system, as well as in single-family housing areas stormwater from the impervious area is discharged into the soil. In Lodz, the concentrations of heavy metals are monitored only in the effluent of the WWTP. According to the current legislation, the stormwater discharged into the environment must meet only the requirements for the concentration of suspended solids and hydrocarbons.

**Table 2:** Catchments of separate storm sewer system in Lodz

River	Location	Land use (order of decreasing share of surface)	Total area (ha)	Coeff. of imperviousness
Olechówka River	south	MFR, SFR, IND, R	2400	0.42
Ner River	southeast	G, IND, R, SFR	1130	0.39
Jasień River	east	IND, R, SFR, MFR IND,	990	0.47
Łódka River	northwest	R, SFR, G	890	0.41
Sokołówka River	north	IND, R, SFR	580	0.43
Zimna Woda River	northwest	IND, R, MFR, SFR	190	0.40
Aniołówka River	northwest	IND, R	170	0.60
Jasieniec River	west	MFR, IND, R	170	0.48
Wrząca River	north	MFR, IND, R, SFR	150	0.47
Others			110	
Total			6800	0.43

*IND* industrial, *R* roads, *C* commercial, *SFR* single-family residential, *MFR* multi-family residential, *G* green

Rivers in Lodz are the receivers of discharges from separate storm sewer system (SS), CSOs, and wastewater treatment plant. Some stretches of the rivers in the city were regulated, riverbed were paved and covered, especially in the city center, where combined sewer overflows function. In areas equipped with separated system rivers collect untreated stormwater runoff, their ecological status also is not good. Rivers and existing reservoirs on them, in many cases lost their recreational and esthetic value for inhabitants of the city.

Volumes of discharges in the research period are presented in Table 3. For CSOs and WWTP, they were established based on data obtained from the operator of the sewer system and WWTP. Volume of discharges from SS was calculated based on catchment area, the coefficient of imperviousness, and total annual precipitations in the period 2011–2013 (respectively 483, 521, and 635 mm).

**Table 3:** Volume of annual discharges into rivers from SS, CSOs, and WWTP in 2011–2013 (thousand m^3^)

Year	SS	CSO	WWTP
2011	13,160	1400	70,560
2012	14,200	807	63,760
2013	17,300	870	69,770

The city has taken actions to restore river basins and reservoirs where this is still possible. They consist of restoring the natural state of the valleys of the rivers through their regulation by natural methods and restoration/rehabilitation of water bodies. The condition for successful river restoration, however, is the limitation of pollutants inflow, especially potentially toxic to aquatic life, like heavy metals. Research conducted by Urbaniak et al. (2008) on one of the Sokolowka reservoirs showed that the concentration of heavy metals (Ni, Cu, Mo, Co, Cd, Pb, Mn, As, and Se) was higher than that present in the reservoir in an agricultural catchment. Further research indicated that stormwater runoff might present a toxic threat on aquatic ecosystems, and winter was the period with the highest risk of toxicity, especially during snowmelt (Szklarek et al. 2015).

### Methods of research

The research on the quality of stormwater from the surface of the different land use and wastewater discharges to surface waters was carried out in Lodz in 2011–2013. In particular, the research has been conducted on:

- runoffs from roofs covered with various roofing, especially those of heavy metals, such as galvanized steel and copper sheet,
- runoffs from roofs with metal elements after the passing through vegetated soil,
- runoffs from streets and parking lots in the city center and areas outside the central districts,
- outlets from SS into urban rivers,
- outlets from the stormwater settling tank,
- CSO discharges, and
- treated wastewater from WWTP.

The data from three CSOs, six outlets from SS, and WWTP outflows were taken into account for balancing loads of heavy metals emitted from the catchment area into receiving waters. Other findings were the basis for the discussion of the main sources of pollution and possibilities of their limitations. The areas of the examined combined catchment were J1–211 ha, J4–1714 ha, B1–610 ha. These CSOs characterize high frequency of activation and discharge the most volume of wastewater into receivers. The tested outlets from SS were located on catchments of a different size of surface and way of land use. Sampling site (outlets from SS and CSOs) are presented on Fig. 1. Samples were collected during all events at intervals of 5–20 min depending on the sewage flow rate. At the same time the volume of wastewater discharged to the receiver was measured. In all the samples, the concentration of four metals zinc, copper, lead, and cadmium were determined using atomic absorption spectrophotometry method (AAS, SOLAAR M5, Thermo Electron Corporation) after digestion with 65% HNO_3_ in microwave digestion (MARSXpress by CEM Company). All analyses were conducted in accordance with the Polish and European Standards. The flame module was used for Zn and Cu, and the graphite furnace module for Pb, Cd, and Cu (for lower concentrations). Limit of detection was 10 μg/l for Zn, 0.5 μg/l for Cu, 1 μg/l for Pb, and 0.1 μg/l for Cd. The event mean concentration of heavy metals (EMC) for an individual event was calculated as follows:

EMC *=* $$ \frac{\sum_{i=1}^n{c}_i{V}_i}{\sum_{i=1}^n{V}_{i.}} $$

*c*_*i*_—concentration in the sample taken during the period *i*

*V*_*i*_—volume of flow during the period *i*

*n*—total number of samples taken during the event

Statistical analysis of obtained data and determining the Pearson’s correlation coefficients between tested metals were carried out with the use of the *Statistica* 10.0 software.

The results of wastewater quality examinations were also obtained from WWTP (inlet and outlet). At the same time, the rainfall intensity in the city was measured using three own rain gauges, and the urban rain gauges network consisted of 18 stations. Continuous measurements of the volume of CSO discharges were carried out by the operator of the sewer system (Sakson et al. 2014).

## Results and discussion

### Analysis of research results

Research was carried out during different rain events, characterized by rainfall amount *H* (mm), total duration *D* (min.), mean and maximum intensity (*i*_min_ and *i*_max_, mm h^−1^), and antecedent dry period ADP (day) (Table 4).

**Table 4:** Range of rain events parameters

Sampling site	*H* (mm)	Duration (min)	*i*_mean_ (mm h^−1^)	*i*_max_ (mm h^−1^)	ADP (day)
CSOs (*n* = 10)	3.2–36.2	15–535	1.2–14.1	3.7–52.8	1–120
Separate system (*n* = 12)	3.3–36.2	30–630	1.5–33.2	3.4–165.5	1–9

The results of analysis of heavy metals concentrations are shown in Table 5. The obtained data indicate a large diversity in the concentrations of heavy metals depending on the place of sampling, but, as in other studies (Brombach and Fuchs 2001; Gasperi et al. 2012; Zgheib et al. 2012), in all samples, zinc was present in the highest concentrations, followed by copper, lead, and lastly cadmium.

**Table 5:** The ranges of heavy metals concentrations (μg/l) in stormwater runoff in Lodz in comparison with rainwater and permissible concentrations of heavy metals in water and sewage in Poland

Sampling site	Zn	Cu	Pb	Cd
Rainwater	30–80	< 0.5–40	< 1–16	< 0.1
Roofs without metal elements	50–1060	< 0.5–133	1–108	< 0.1–2.1
Roofs with metal elements	520–31,300	7–6993	1–60	0.1–2.5
Roofs with metal elements—after the passing through vegetated soil	30–2280	< 0.5–850	2–29	0.1 < − 1.9
Streets and parking lots	80–4180	28–297	< 1–130	< 0.1–35.7
Outlets from separate storm sewers into urban rivers	40–1060	23–95	10–126	< 0.1–0.6
Outlets from the stormwater settling tank	50–160	11–20	11–20	< 0.1
CSO discharges	170–1890	31–590	12–300	< 0.1–2.9
Treated wastewater from GWWTP	70–150	20–40	< 1	< 0.1
Permissible concentrations				
Industrial wastewater discharged into receiving water	2000	100–500	100–500	50–400
Surface water of good quality	1000	50	7.2	0.25 (annual average) (max.) 5
Drinking water	–*	2000	25	

*Not defined in Polish regulations; level recommended by WHO is 5000 μg/l

The highest concentrations of heavy metals were observed in roof runoff from roofs with metal elements (roofing material, gutters), especially of zinc and copper. They were sometimes several times higher compared to stormwater from urban drainage systems. Similar results were obtained in other studies (Davis et al. 2001; Gromaire et al. 2001; Göbel et al. 2007). High concentrations of zinc were also sometimes observed in runoff from street and parking lots, which is probably related to the leaching of metals from vehicles, safety fences, elements, and metallic structures of streets and parking lots. Huber et al. (2016) who analyzed dataset of 294 monitored sites found that Zn concentrations were very variable in traffic area runoff compared with other heavy metals because of its presence in galvanized structures and crumbs of car tire rubber and no historical trend is detected for Zn. Historical trends for Pb in these runoff show a sharp decrease during recent decades, as a result of elimination of leaded gasoline. The median total Pb concentrations of the twenty-first century for North America and Europe are approximately 15 μg/l. A similar value was observed in Lodz; although in the last decade, the number of cars in the city increased by more than 50%.

In runoffs, discharged into urban rivers from separate system large differences of heavy metals concentrations, especially zinc were found. For residential catchments, concentrations were relatively low, while in the case of industrial catchments and areas of large share of traffic area were the highest. Large variation of heavy metals concentration was also observed between different rainfall events at the same sampling site. As Huber et al. (2016) noticed, a combination of several interacting factors results in heavy metal runoff pollution, and these factors should be described in detail for each monitoring site. In addition to the land use and point pollutants source, the parameters of precipitation have a decisive influence on the dynamics of pollutants emission to the receiver. The antecedent dry period have a fundamental significance for the amount of pollutants accumulated on the catchment and washed-off during rainfall phenomenon. In Lodz, the research was conducted during rainfall with very diverse characteristics, which allowed to assess the variability of heavy metals concentrations in outflows from each catchments.

As limits of metals in the stormwater discharged into surface waters are not defined in Polish legal regulations, the research results can be compared only to the limits for drinking water in accordance with the Regulation of the Minister of Health, the requirements for industrial wastewater discharged into receiving waters and soil in accordance with the Regulation of the Minister of Environment, as well as with the criteria of evaluation of surface waters in accordance with the Regulation of the Minister of Environment. As can be observed, concentrations of heavy metals often exceed the permitted levels for industrial wastewater discharged into receiving waters and limits for surface water of good quality (Table 5).

**Fig. 2:**
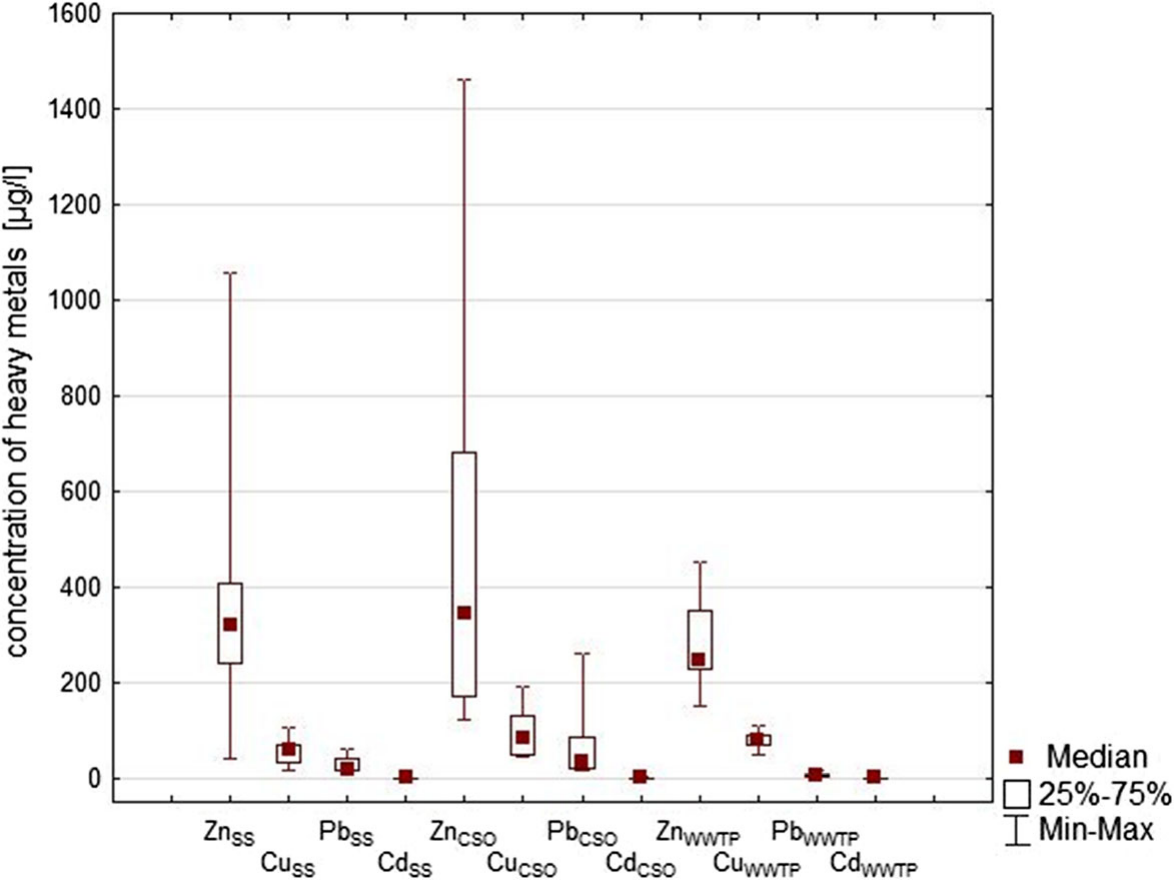
Content of heavy metals in wastewater from Lodz catchment (EMC in discharges from SS, and CSOs, average flow-weighted concentration in inflow to WWTP)

Figure 2 present the results of heavy metals EMC for studied rainfalls; the data were compared with other studies in Table 6. In Fig. 2, concentrations of metals in wastewater inflow to WWTP are also presented.

**Table 6:** The median of EMC of heavy metals (μg/l) in outlets from SS and CSOs in comparison with other studies

	Zn	Cu	Pb	Cd
Outlets from separate storm sewers	320	60	15	0.5
References				
Brombach and Fuchs (2001)	1–3565	3–1800	0.2–2745	0.3–37
O’Sulivan et al. (2012)	271.0	16.0	26.0	–
Zgheib et al. (2012)	130–520	30–220	< 10–129	–
Gasperi et al. (2014)	126–240	15–138	2.5	0.3
CSO discharges	345	84	35	0.2
Reference*s*				
Brombach and Fuchs (2001)	41–1548	40.8–510	17–320	0.3–25
Irvine et al. (2005)	676	24.4	90.2	10.2
Gasperi et al. (2008)	248–3525	38–1180	10–117	< 1–2.4
Gasperi et al. (2012)	658–1137	86–134	46–175	–

As can be seen the concentrations of heavy metals measured in the Lodz city were in the ranges of results from other studies. The data from literature presented in Table 6 show a very large variation of concentrations in the individual sampling sites. For example, Zgheib et al. (2012) who examined priority pollutants in stormwater in Paris highlighted that concentrations of Zn, Cu, and Pb were twice as high as those for stormwater in London (Rule et al. 2006). In turn, research conducted by Gasperi et al. (2014) on three urban catchments has shown that for many pollutants, there were no significant differences in stormwater quality; but for several metals, significant differences were observed.

Heavy metals concentrations are usually higher in combined sewage than in sanitary (Brombach and Fuchs 2001; Irvine et al. 2005, Gasperi et al. 2012). Stormwater is recognized as the most important source of heavy metals in urban catchment (Davies et al. 2001, Gasperi et al. 2010; Barbosa et al. 2012), and thus it usually cause an increase of heavy metals concentration in combined flow during the wet weather. Kafi-Benyahia et al. (2005) observed that Cu had a higher concentration in wet weather flow than in dry weather flow, and runoff, but Cd, Pb, and Zn, were higher concentrations in urban runoff than in wet weather flow and dry weather flow. Discharges of untreated stormwater and wastewater from CSOs can be particularly hazardous for small urban streams but the threat depends on many factors, i.e., bioavailability of metals (Angerville et al. 2013; Kominková et al. 2016).

Concentrations of heavy metals in inflow to WWTP (Fig. 2) were lower than levels in CSOs and outlets from SS, and were variable, probably mainly due to the fact, that WWTP supports both sanitary and combined sewer system. Additionally, as Rule et al. (2006) observed, concentration of heavy metals entering an urban wastewater catchment may vary considerably over the course of the week. Many heavy metals occurring in wastewater may have toxic effects on aquatic life and can inhibit biological processes at the WWTP. According to Coello Oviedo et al. (2002), cadmium is the most highly toxic metal for the microbial communities present in the activated sludge process, followed by copper, and lastly zinc. However, the negative effects of the presence of heavy metals in the wastewater influent are observed at higher concentrations than those found in the WWTP in Lodz. Average flow-weighted concentrations of heavy metal in outflow from WWTP were low: Zn 59–88 μg/l, Cu < 50 μg/l, Pb < 4 μg/l, and Cd < 0.5 μg/l. Wastewater treatment plants greatly reduce total metal concentrations but only slightly reduce labile metal concentrations (Buzier et al. 2011). For example, the global removal yield of monitored at the Saine-Aval WWTP was not less than 75% for total Cu and Pb concentration. However, dissolved and labile metal removal was lower and more dependent on the plant operating conditions. Treated urban wastewater discharges may be thus a source of bioavailable trace metals (Buzier et al. 2006).

Analysis of Pearson’s correlation coefficients showed that strong positive correlations occur only between Zn and Pb in CSOs discharges and inflows to WWTP, as well as between Zn and Cd, and Pb and Cd in CSOs discharges. In the case of studied individual outlets of separate system, some correlations were also significant; but in general for stormwater, no strong correlations between the tested metals were found (Table 7).

**Table 7:** Pearson’s correlation coefficients for heavy metals concentrations in wastewater from SS, CSOs, and WWTP

	SS				CSO				WWTP			
	Zn	Cu	Pb	Cd	Zn	Cu	Pb	Cd	Zn	Cu	Pb	Cd
Zn		0.34	0.28	0.06		0.19	*0.91*	*0.89*		0.67	*0.79*	0.66
Cu	0.34		0.34	0.66	0.19		0.14	0.10	0.67		0.69	0.48
Pb	0.28	0.34		0.35	*0.91*	0.14		*0.99*	*0.79*	0.69		0.74
Cd	0.06	0.66	0.35		*0.89*	0.10	*0.99*		0.66	0.48	0.74	

Strong positive correlation was mark in italic

### Loads of heavy metals emitted into receiving waters

For catchment area, both with separate and combined sewer systems, the unitary loads of discharged heavy metals were calculated. The data were compared with the results of other research in Table 8.

**Table 8:** Annual mass loads of heavy metals per unit of area (g/ ha /year) in comparison with other studies

Location	Zn	Cu	Pb	Cd
Separate system	700	130	33	0.9
CSO	84	20	8	0.5
References				
Wong et al. (1997)	600	200	600	–
Davis et al.(2001)	646	38	69	1.2
Birch and Rochford (2010)	378	72	82	11
O’Sullivan et al. (2012)	1038	62	100	–
Wang et al. (2013) campus area	100	240	330	40
Wang et al. (2013) urban city road	6000	1240	6300	500
Huber and Helmreich (2016) traffic area (median of 45 sites)	1960	355	110	6.8
Järveläinen et al. (2017) city center	650–2100	160–890	40–2010	–
Järveläinen et al. (2017) single-family res.	40–430	10–80	1–190	–

**Fig. 3:**
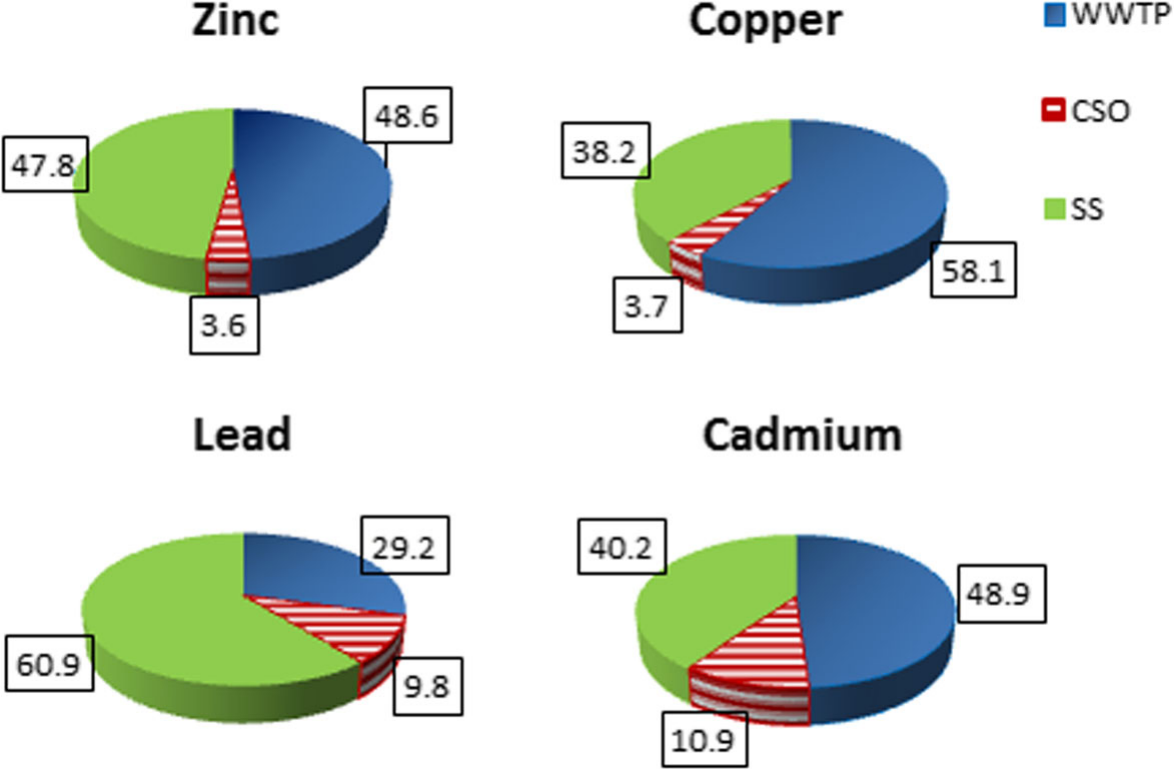
Percentage of outlets from SS, CSOs, and WWTP in the overall load of heavy metals discharged from the catchment in Lodz

In case of the outlet from WWTP due to the limited level of accuracy of the determinations of the metals concentration in the effluent, the load was calculated assuming 75% reduction of pollution. The outlets from wastewater treatment plants are best studied and controlled source of pollution, but as can be seen in the case of heavy metals, they are responsible for approx. 50% of the pollution load of zinc, cooper, and cadmium and only 30% of lead, which over 60% comes from separate system (Fig. 3). The analysis shows that the untreated stormwater is the most important source of heavy metals discharged into the environment from the urban catchment. For example, during a 4-h precipitation of 11-mm total depth, about 6.5 kg of Zn and 1.85 kg of Cu were discharged to the river from a residential catchment area of 340 ha, while the average daily loads discharged from WWTP were 13 kg of Zn and 3.7 kg of Cu. Research conducted in Paris by Kafi et al. (2008) allowed to estimate heavy metal loads at 0.10 g ha active^−1^ for Cd, 13 g ha active^−1^ for Cu, 11 g ha active^−1^ for Pb, and 98 g ha active^−1^ for Zn; but these loads exhibit high variability from rain event to event. It should be noted that in case of separate sewer system, a comparable or larger annual pollution load is discharged into the rivers in a shorter time, sometimes in a violent manner, as in the case of combined sewer overflows.

As other studies demonstrated, the annual mass loads of heavy metals per unit of area varied widely depending on the location and land use of the catchment. It can be seen, for example in research conducted by Järveläinen et al. (2017). The annual mass load of pollutant is often analyzed with reference to atmospheric deposition. According to research conducted by Sabin et al. (2005), total deposition flux: wet + dry (mg/m^2^/year) was Zn 14.5, Cu 3.4, Pb 2.0, and atmospheric deposition potentially accounted for as much as 57–100% of the total trace metal loads in stormwater. Wet deposition comprised 1–10% of the total annual deposition. In Lodz region, atmospheric deposition of heavy metals in 2011–2013 (Report 2015) was higher for zinc—about 50 mg/m^2^/year; coper—8 mg/m^2^/year; and lower for lead—1 mg/m^2^/year. It was from 30% (for Pb) to 70% (for Zn) of the load of heavy metals in stormwater discharged from the area equipped with separate system. Research has shown (Report 2015) that in the years 1999–2015 atmospheric deposition of lead and also cadmium in the Lodz region steadily decreased which certainly has an impact on reducing the load of heavy metals discharged with stormwater.

### Possibilities of heavy metals’ load limitation

Identification of the most important sources of pollutants, particularly potentially toxic for receiving waters, is required in effective stormwater management in urban areas. The achievement of good water status in rivers and reservoirs in Lodz in accordance with the WFD requires, among other, limiting loads of heavy metals discharged from urban catchment. The type of sewage system in the catchment (separate or combined) plays an important role in the discharges of heavy metals to the receiving aquatic environment (Becouze-Lareure et al. 2016; Kominková et al. 2016) and mixed system of sewerage in Lodz poses many problems which should be considered for an effective control of heavy metal loads**.** Summarizing, the results of the research show that:

- The main issue in the city is high emission of heavy metals from separate system, especially zinc and copper. Concentrations and loads of lead and cadmium are much lower and systematically decrease. Most metals present in stormwater are primarily bound to the particulate phase (Gasperi et al. 2014) and they can be easily removed by sedimentation, which was also confirmed in this study (Table 5). The high concentration of total suspended solids in stormwater in Lodz (Zawilski and Sakson 2013) indicates the need for the construction of the treatment facilities on some of the biggest outlets. However, other solutions that meet the requirements of best management practices (BMP)/low impact development (LID) should be also considered (Zawilski et al. 2014). The highest concentrations of zinc and cooper were observed in runoff from roofs with metal elements, streets, and parking lots. These runoffs should be managed in local BMP/LID implementations. Contaminants contained in urban stormwater may be removed during infiltration through porous media systems and in bioretention systems. The removal of heavy metals in infiltration and bioretention systems varies from 55 to 99% (Langeveld et al. 2012; Grebel et al. 2013). Research conducted in Lodz has shown, that efficiency of zinc and cooper removal from roof runoffs in vegetated soil is very high (over 90%) regardless of the initial concentration (Sakson 2017). To reduce the risk of groundwater contamination plants used in phytoremediation can be applied additionally, although garden grass is also effective. Another efficient method may be adsorption. The level of metal removal by adsorption depends on the type and concentration of metal as well as the filter material (Reddy et al. 2014). Maximum removal rates achieved for Cd, Cu, Pb, and Zn by calcite, zeolite, and iron filings in conducted research were 95–100%. In choosing optimal stormwater treatment methods fractionation of heavy metals on given site must be considered, for example in road runoff Pb are mostly particle-bound, while Zn, Cu, and Cd occur at a higher fraction in the dissolved phase (Huber et al. 2016). In the last decades, source control technologies were more used than conventional systems of stormwater treatment (Barbosa et al. 2012). They have such advantages like easier implementation, greater private investment and benefits, and potential microclimate benefits, but also drawbacks like limited volume treated and uncertain maintenance regimes (Fletcher et al. 2013). Hence, all aspects should be taken into account before making decision concerning stormwater management in individual catchment.
- Effective reduction of heavy metals loads discharged into receiving water and mitigation of environmental negative impacts requires knowledge of sources and emissions for each catchment. Furthermore, it must be established whether the whole volume of stormwater runoff should be treated, or just the first flush runoff. The determination that the first flush control is enough effective reducing receiving waters impact would limit the volume of the purification facilities and optimize the costs of stormwater management. Usually the runoff is characterized by highly variable concentrations of pollutants including heavy metals during rain phenomenon. Most often, greatest load of pollutants flows at the beginning of the phenomenon, but the findings made on some catchments indicate that in the case of some rainfall the highest levels of contamination appear on the runoff end. Therefore, the use of event mean concentration can lead to making wrong decisions as to the method of runoff treating and facilities dimensioning. Additionally, in temperate climates seasonal variability of metal load emissions are observed and should be taken into account in planning. Tiefenthaler et al. (2008) found that metals loadings from early season storms were higher than from late season storms. In turns, research conducted by Blecken et al. (2012) indicates that relatively high amounts of contaminants are discharged during snowmelt.
- In the case of the use of treatment facilities on the outlets of sewage system, additionally, the possibility of interactions and transformations of pollutants in sewers must be taken into account. According to Lundy et al. (2012), there is considerable opportunity for pollutant transformations during conveyance from the original source to the final discharge point. Research conducted by Gromaire et al. (2001) in Paris has shown a change of the chemical form of heavy metals during the transport in the sewer. For cadmium, copper, and zinc the mass of particle-bound metals increased between the entry and the exit of the combined sewer. Gasperi et al. (2010) observed a large loss of dissolved metals during the transfer within the sewer network. Therefore, optimal selection of treatment methods and facilities requires more detailed research focused on the nature of heavy metals form and the relationship between heavy metal concentrations and particle-size distributions. There is also a need to assess the impact of the type of contaminants on receiver. Lundy et al. (2012) highlighted that identification of pollution sources posing the greatest level of risk to receiving water quality plays important role in decision-making process with regard to the optimal allocation of infrastructure investments.
- Reducing the amount of heavy metals discharged into the receivers by combined sewer overflows will occur together within the limit of frequency of CSO activities in the city in accordance with legislation requirements. This problem is being solved in the city by disconnecting drainage system on some catchments and by construction of detention tanks. The efficiency of regulating weirs of CSOs was also analyzed (Brzezinska 2017). Using this type of solutions results that greater volume of wet weather flow is directed to the treatment plant, in which the effective heavy metals’ removal takes place. The possibility of reducing the impervious surface connected to the combined sewer system was also analyzed. However, under the conditions of the densely built-up urban catchment, disconnection of roofs will not allow for significant improvement in the functioning of the drainage system. Analyses made with the use of the Hydra software (Pizer Incorporated Seattle USA) have shown that only single discharges were eliminated in the year and the total volume of discharges was reduced by 15–30% (Zawilski and Sakson 2005). Analysis concerned disconnecting all possible roofs in case of availability of the site for infiltration facilities, which is not possible in under real conditions.
- Other activities and solutions, such as proper maintenance of metal surfaces, especially roofs, or street sweeping can also help to reduce the emissions of heavy metals into the receiving water. According to Gromaire et al. (2001), in Paris, more than 80% of the Cd, Pb, and Zn loads and about 40–85% of Cu in the runoff are attributable to roof runoff, due to the corrosion of the frequent metallic roof coverings and roof fittings. Hence, stopping the use of metal roofing or their proper maintenance could effectively reduce emission of heavy metals. However, in the case of sweeping, according to Kang et al. (2009), there is little quantitative evidence that street sweeping directly improves runoff water quality. The highest concentrations of heavy metals are in fines fractions, but street sweepers are more effective in removing coarse sediments than fine (German and Svensson 2002). So, there is a need to investigate the particle-size distribution and metal content in swept sediments to determine the real effectiveness of street sweeping.
- Metal loads from urban areas can be predicted using a rainfall-runoff model. Build-up- and wash-off processes may be analyzed, for example, with the use of US EPA Stormwater Management Model (SWMM), but it should be calibrated on hydrological data, rainfall intensity, and stormwater quality (Yuan et al. 2001; Wicke et al. 2012). The use of mathematical models, however, can give a more complete assessment of emissions and limits the lengthy and costly sampling and research works. They can be also useful in predicting changes resulting from the development of the city and climate change. Borris et al. (2016) who assessed the future trends in urban stormwater quality (TSS and three heavy metals: zinc, copper, and lead) highlighted that pollutants’ loads were more sensitive to changes in socio-economic factors (i.e., increasing traffic intensities, growth, and intensification of the individual land uses) than to climate changes.

Based on conducted research, literature data, and analysis of the functioning of the sewer system carried out earlier with the use of the US EPA SWMM and rainfall data (Brzezinska 2017; Sakson et al. 2017), the possibilities of heavy metals’ loads reducing in receiving waters were assessed (Table 9). It was assumed that stormwater would be treated in centralized facilities like settling tanks and wetlands or local BMPs/LIDs (infiltration facilities or rainwater harvesting and reuse) depending on local conditions. As regards CSOs and WWTP possible reduction of heavy metals’ loads was determined assuming the use of detention in existing wastewater system, which will enable limiting the discharge of untreated wastewater into the receiver in accordance with legal regulations.

**Table 9:** Possibility of reducing of heavy metals’ loads discharged into receiving waters in Lodz city

Solution	Method of assessment	Efficiency (%)			
		Zn	Cu	Pb	Cg
Construction of stormwater treatment facilities on main outlets of stormwater drainage (e.g. settling tanks, wet ponds, constructed wetlands, detention or retention ponds)	Analysis of the catchment and sewage system.	20.1	16.0	25.6	16.9
	It was assumed the construction of 7 settling tanks on the largest catchments (mainly industrial and multi-family residential).				
	Drained area—4000 ha.				
	Efficiency of heavy metals removal according to conducted research and literature data ~ 70% (60–90%)				
Local BMPs/LIDs on other catchments	It was assumed the BMPs/LID implementation for runoffs from 50% surface of roofs, streets and parking	9.6	7.6	12.2	8.0
(infiltration facilities or rainwater harvesting and reuse)	lots, mainly in single-family residential area Total catchment area—2800 ha				
CSOs attenuation (detention tanks, weirs’ regulation)	Analysis of CSOs’ functioning with the use of US	0.9	0.9	2.5	2.7
	EPA SWMM. Reduction of discharge volume (while limiting the number of overflows to 10 per year according to formal requirements) ~ 25% (10–30% for the period of 10 years)				
Improve the efficiency of treatment in WWTP by detention of wet weather flow (storage tanks, in-sewer storage)	Analysis of detention usage in limiting flow to WWTP with the use of US EPA SWMM.	0.9	1.1	0.5	0.9
	At present biologically untreated wastewater ~ 3% of total inflow.				
	Annual reduction of the volume of biologically untreated wastewater—70%.				

As can be seen in Table 9 the most efficient solution is treatment of stormwater runoff on the outlets of separate sewer system, but the use of local BMPs/LID is also effective option. These solutions can limit pollutants load discharged from urban catchment with stormwater and also can contribute to the reduction of stormwater discharged into the combined sewer system in the central districts, and thus to limit the CSOs functioning. CSOs’ attenuation by detention tanks or weirs’ regulation is not very effective because eliminates discharges of the smallest volume.

## Conclusions

Effective protection of water in the urbanized catchment equipped with two types of sewage system, combined with CSOs and separate, requires monitoring and reducing pollutant emissions from many sources: point and diffuse non-point. In order to assess the receiver threat, it is necessary not only to identify the loads of potentially harmful substances discharged to the aquatic environment, but also to determine the dynamics of their emissions. In the case of WWTPs pollutants are released to the receiver in limited amounts in a continuous manner, in the case of CSOs in a violent manner at high concentrations, similarly, although slightly less rapidly this is in the case of stormwater discharges. The knowledge gained as a result of the qualitative and quantitative monitoring of discharges into receiving waters allows to make optimal decisions about technical solutions to reduce emissions, such as storage tanks, treatment facilities, BMPs/LIDs, and about other activities not directly related to stormwater management, like street sweeping or reducing the use of products containing toxic substances, for example heavy metals. Quality and quantity characteristic of discharges into receiving waters is one of the most important factors that should be recognized in sustainable stormwater management in urban areas. Data obtained from monitoring enable optimal use of funds for this purpose.

The condition for the improvement of water quality in rivers and reservoirs in Lodz city is, among others, reducing the amount of heavy metals in discharges from urban catchment, in particular metals contained in stormwater runoff. The level of emissions of heavy metals from the catchment is not higher than in other cities, but due to the fact that in Lodz small rivers are the receivers of sewage, the reduction of this emission is particularly important. The research on stormwater quality and the measurement of precipitations and flow for CSOs and the Group Wastewater Treatment Plant carried out in Lodz allowed for recognizing surfaces and catchments from which the runoff is characterized by the highest concentrations of heavy metals and for the assessment of their load discharged into receiving waters from separate system, CSOs and the WWTP. Studies have shown that future efforts in the city should be primarily focused on limitation of discharges of untreated stormwater into receiving waters. Stormwater treatment facilities should be constructed on the main outlets of stormwater drainage. Implementation of well-design BMPs/LIDs is needed in the city for management of stormwater runoff from roofs, streets and parking lots. Optimal stormwater management and effective protection of receiving waters require further research, more detailed and focused on sources on a catchment scale. Future studies concerning the forms of heavy metals and their impact on receiving water and toxicity are also necessary.

## References

[CR1] Angerville R, Perrodin Y, Bazin C, Emmanuel E (2013). Evaluation of ecotoxicological risks related to the discharge of combined sewer overflows (CSOs) in a Periurban River. International Journal of Environmental Research and Public Health.

[CR2] Barbosa AE, Fernandes JN, David LM (2012). Key issue for sustainable urban stormwater management. Water Research.

[CR3] Becouze-Lareure C, Dembélé A, Coquery M, Cren-Olive C, Barillon B, Bertrand-Krajewski J-L (2016). Source characterization and loads of metals and pesticides in urban wet weather discharges. Urban Water Journal.

[CR4] Birch GF, Rochford L (2010). Stormwater metal loading to a well-mixed/stratified estuary (Sydney Estuary, Australia) and management implications. Environmental Monitoring and Assessment.

[CR5] Blecken G-T, Rentz R, Malmgren C, Öhlander B, Viklander M (2012). Stormwater impact on urban waterways in a cold climate: variations in sediment metal concentrations due to untreated snowmelt discharge. Journal of Soils and Sediments.

[CR6] Blumensaat F, Staufer P, Heusch S, Reußner F, Schütze M, Seiffert S, Gruber G, Zawilski M, Rieckermann J (2012). Water quality-based assessment of urban drainage impacts in Europe—where do we stand today?. Water Science and Technology.

[CR7] Borris M, Leonhardt G, Marsalek J, Österlund H, Viklander M (2016). Source-based modeling of urban stormwater quality response to the selected scenarios combining future changes in climate and socio-economic factors. Environmental Management.

[CR8] Brombach, H., & Fuchs, S. (2001). Datenpool gemessener Verschmutzungskonzentrationen von Trocken- und Regenwetterabflüssen in Misch- und Trennkanalisationen, Abschlussbericht.

[CR9] Brown JN, Peake BM (2006). Sources of heavy metals and polycyclic aromatic hydrocarbons in urban stormwater runoff. Science of the Total Environment.

[CR10] Brzezinska A (2017). Impact assessment of combined sewer overflows regulation on sewage volume discharged into surface waters—a case study. Ochrona Środowiska (Environmental Pollution Control).

[CR11] Buzier R, Tusseau-Vuillemin M-H, Martin dit Meriadec C, Rousselot O, Mouchel J-M (2006). Trace metal speciation and fluxes within a major French wastewater treatment plant: impact of the successive treatments stages. Chemosphere.

[CR12] Buzier R, Tusseau-Vuillemin M-H, Keirsbulck M, Mouchel J-M (2011). Inputs of total and labile trace metals from wastewater treatment plants effluents to the Seine River. Physics and Chemistry of the Earth.

[CR13] Coello Oviedo MD, Sales Márquez D, Quiroga Alonso JM (2002). Toxic effects of metals on microbial activity in the activated sludge process. Chemical and Biochemical Engineering Quarterly.

[CR14] Davis AP, Shokouhian M, Ni S (2001). Loading estimates of lead, copper, cadmium, and zinc in urban runoff from specific sources. Chemosphere.

[CR15] EC (2000). EU Water Framework Directive: Directive 2000/60/EC of the European Parliament and of the Council establishing a framework for Community action in the field of water policy. Official Journal of the European Communities, L.

[CR16] EC (2008). Directive 2008/105/EC of the European Parliament and of the Council of 16 December 2008 on environmental quality standards in the field of water policy, amending and subsequently repealing Council Directives 82/176/EEC, 83/513/EEC, 84/156/EEC, 84/491/EEC, 86/280/EEC and amending Directive 2000/60/EC of the European Parliament and of the Council. Off. J. Eur. Communities, L.

[CR17] Eriksson E, Baun A, Scholes L, Ledin A, Ahlman S, Revit M, Noutsopoulos C, Mikkelsen PS (2007). Selected stormwater priority pollutants—a European perspective. Science of the Total Environment.

[CR18] Fletcher TD, Andrieu H, Hamel P (2013). Understanding, management and modelling of urban hydrology and its consequences for receiving waters: a state of the art. Advances in Water Research.

[CR19] Gasperi J, Garnaud S, Rocher V, Moilleron R (2008). Priority pollutants in wastewater and combined sewer overflow. Science of the Total Environment.

[CR20] Gasperi J, Gromaire MC, Kafi M, Moilleron R, Chebbo G (2010). Contributions of wastewater, runoff and sewer deposit erosion to wet weather pollutant loads in combined sewer systems. Water Research.

[CR21] Gasperi J, Zgheib S, Cladière M, Rocher V, Moilleron R, Chebbo G (2012). Priority pollutants in urban stormwater: Part 2—case of combined sewers. Water Research.

[CR22] Gasperi J, Sebastian C, Ruban V, Delamain M, Percot S, Wiest L, Mirande C, Caupos E, Demare D, Diallo Kessoo Kessoo M, Saad M, Schwartz JJ, Dubois P, Fratta C, Wolff H, Moilleron R, Chebbo G, Cren C, Millet M, Barraud S, Gromaire MC (2014). Micropollutants in urban stormwater: occurrence, concentrations, and atmospheric contributions for a wide range of contaminants in three French catchments. Environmental Science and Pollution Research.

[CR23] German J, Svensson G (2002). Metal content and particle size distribution of street sediments and street sweeping waste. Water Science and Technology.

[CR24] Göbel P, Dierkes C, Coldewey WG (2007). Storm water runoff concentration matrix for urban areas. Journal of Contaminant Hydrology.

[CR25] Grebel JE, Mohanty SK, Torkelson AA, Boehm AB, Higgins CP, Maxwell RM, Nelson KM, Sedlak DL (2013). Engineered infiltration systems for urban stormwater reclamation. Environmental Engineering Science.

[CR26] Gromaire MC, Garnaud S, Saad M, Chebbo G (2001). Contribution of different sources to the pollution of wet weather flows in combined sewers. Water Research.

[CR27] Huber H, Helmreich B (2016). Stormwater management: calculation of traffic area runoff loads and traffic related emissions. Water.

[CR28] Huber M, Welker A, Helmreich B (2016). Critical review of heavy metal pollution of traffic area runoff: occurrence, influencing factors, and partitioning. Science of the Total Environment.

[CR29] Irvine KN, Caruso J, Mccorkhill G (2005). Consideration of metals levels in identifying CSO abatement options. Urban Water Journal.

[CR30] Järveläinen J, Sillanpääb N, Koivusalo H (2017). Land-use based stormwater pollutant load estimation and monitoring system design. Urban Water Journal.

[CR31] Joshi UM, Balasubramanian R (2010). Characteristics and environmental mobility of trace elements in urban runoff. Chemosphere.

[CR32] Kafi M, Gasperi J, Moilleron R, Gromaire MC, Chebbo G (2008). Spatial variability of the characteristics of combined wet weather pollutant loads in Paris. Water Research.

[CR33] Kafi-Benyahia M, Gromaire MG, Chebbo G (2005). Spatial variability of characteristics and origins of urban wet weather pollution in combined sewers. Water Science and Technology.

[CR34] Kang J-H, Debats SR, Stenstrom MK (2009). Storm-water management using street sweeping. Journal of Environmental Engineering.

[CR35] Karlavičienė V, Švedienė S, Marčiulionienė DE, Randerson P, Rimeika M, Hogland W (2009). The impact of storm water runoff on a small urban stream. Journal of Soils and Sediments.

[CR36] Kominková D, Nábělková J, Vitvar T (2016). Effects of combined sewer overflows and storm water drains on metal bioavailability in small urban streams (Prague metropolitan area, Czech Republic). Journal of Soils and Sediments.

[CR37] Langeveld JG, Liefting HJ, Boogaard FC (2012). Uncertainties of stormwater characteristics and removal rates of stormwater treatment facilities: implications for stormwater handling. Water Research.

[CR38] Lundy L, Ellis JB, Revitt DM (2012). Risk prioritization of stormwater pollutant sources. Water Research.

[CR39] Ma Y, Egodawatta P, McGree J, Liu A, Goonetilleke A (2016). Human health risk assessment of heavy metals in urban stormwater. Science of the Total Environment.

[CR40] Munch Christensen A, Nakajima F, Baun A (2006). Toxicity of water and sediment in a small urban river (Store Vejlea, Denmark). Environmental Pollution.

[CR41] O’Sullivan A, Wicke D, Cochrane T (2012). Heavy metal contamination in an urban stream fed by contaminated air-conditioning and stormwater discharges. Environmental Science and Pollution Research.

[CR42] Podstawczynska, A. (2010). Air temperature and precipitation in the Lodz region in the last century (in Polish) http://nargeo.geo.uni.lodz.pl/~meteo/ap/pdf/2010_Zabieniec.pdf?page=4.

[CR43] Reddy KR, Xie T, Dastgheibi S (2014). Removal of heavy metals from urban stormwater runoff using different filter materials. Journal of Environmental Chemical Engineering.

[CR44] Report on the state of environment in Lodz Voivodeship 2015 (in Polish).

[CR45] Rule KL, Combe SDW, Ross D, Thornton A, Makropoulos CK, Rautiu R (2006). Diffuse sources of heavy metals entering an urban wastewater catchment. Chemosphere.

[CR46] Sabin LD, Lim JH, Stolzenbach KD, Schiff KC (2005). Contribution of trace metals from atmospheric deposition to stormwater runoff in a small impervious urban catchment. Water Research.

[CR47] Sakson G (2017). Efficiency of heavy metals removal during roof runoff infiltration through vegetated soil. Environment Protection Engineering.

[CR48] Sakson, G., Zawilski M., & Brzezińska, A. (2014). Assessment of heavy metal loads emitted from urban catchment based on Lodz City. *Proc. 13*^*th*^*International Conference on Urban Drainage*, Sarawak, Malaysia, 7–12 September 2014.

[CR49] Sakson, G., Zawilski M, & Brzezińska A. (2017) Analysis of combined sewer flow storage scenarios enabling flow attenuation to wastewater treatment plant. *Ecological Chemistry and Engineering S,* (accepted for publication).

[CR50] Selbig W, Bannerman R, Steven R, Corsi SR (2013). From streets to streams: assessing the toxicity potential of urban sediment by particle size. Science of the Total Environment.

[CR51] Szklarek S, Stolarska M, Wagner I, Mankiewicz-Bocze J (2015). The microbiotest battery as an important component in the assessment of snowmelt toxicity in urban watercourses—preliminary studies. Environmental Monitoring and Assessment.

[CR52] Tiefenthaler LL, Eric D, Stein ED, Schiff KC (2008). Watershed and land use–based sources of trace metals in urban storm water. Environmental Toxicology and Chemistry.

[CR53] Urbaniak M, Zieliński M, Wesołowski W, Zalewski M (2008). PCBs and heavy metals contamination in bottom sediments from three reservoirs of different catchment characteristics. Polish Journal of Environmental Studies.

[CR54] Wang S, He Q, Ai H, Wang Z, Zhang Q (2013). Pollutant concentrations and pollution loads in stormwater runoff from different land uses in Chongqing. Journal of Environmental Sciences.

[CR55] Wicke D, Cochrane TA, O’Sullivan A (2012). Buid-up dynamics of heavy metals deposited on impermeable urban surfaces. Journal of Environmental Management.

[CR56] Wong KM, Strecker EW, Stenstrom MK (1997). GIS to estimate storm-water pollutant mass loadings. Journal of Environmental Engineering.

[CR57] Yuan Y, Hall K, Oldhm C (2001). A preliminary model for predicting heavy metal contaminant loading from an urban catchment. Science of the Total Environment.

[CR58] Zawilski, M., & Sakson, G. (2005). The effectiveness of rainwater infiltration in urbanized areas (in Polish). *Monographs of Environmental Engineering Committee of Polish Academy of Sciences, vol. 3.2.*

[CR59] Zawilski M, Sakson G (2013). Assessment of total suspended solid emission discharged via storm sewerage system from urban areas. Ochrona Środowiska (Environmental Pollution Control).

[CR60] Zawilski M, Sakson G, Brzezińska A (2014). Opportunities for sustainable management of rainwater: case study of Łódź, Poland. Ecohydrology and Hydrobiology.

[CR61] Zgheib S, Moilleron R, Chebbo G (2012). Priority pollutants in urban stormwater: Part 1—case of separate storm sewers. Water Research.

